# Subcellular structure, heterogeneity, and plasticity of senescent cells

**DOI:** 10.1111/acel.14154

**Published:** 2024-03-30

**Authors:** Thais Cardoso Bitencourt, Jose Eduardo Vargas, Andrew Oliveira Silva, Lucas Rosa Fraga, Eduardo Filippi‐Chiela

**Affiliations:** ^1^ Programa de Pós‐Graduação Em Biologia Celular e Molecular Universidade Federal do Rio Grande do Sul Porto Alegre Rio Grande do Sul Brazil; ^2^ Departamento de Biologia Celular Universidade Do Paraná Curitiba Paraná Brazil; ^3^ Faculdade Estácio RS Porto Alegre Rio Grande do Sul Brazil; ^4^ Centro de Pesquisa Experimental Hospital de Clínicas de Porto Alegre Porto Alegre Rio Grande do Sul Brazil; ^5^ Programa de Pós‐Graduação Em Medicina: Ciências Médicas Universidade Federal do Rio Grande do Sul Porto Alegre Rio Grande do Sul Brazil; ^6^ Departamento de Ciências Morfológicas Universidade Federal Do Rio Grande Do Sul Porto Alegre Rio Grande do Sul Brazil; ^7^ Centro de Biotecnologia Universidade Federal do Rio Grande do Sul Porto Alegre Rio Grande do Sul Brazil

**Keywords:** cellular senescence, dynamics, heterogeneity, SASP, subcellular structure

## Abstract

Cellular senescence is a state of permanent growth arrest. It can be triggered by telomere shortening (replicative senescence) or prematurely induced by stresses such as DNA damage, oncogene overactivation, loss of tumor suppressor genes, oxidative stress, tissue factors, and others. Advances in techniques and experimental designs have provided new evidence about the biology of senescent cells (SnCs) and their importance in human health and disease. This review aims to describe the main aspects of SnCs phenotype focusing on alterations in subcellular compartments like plasma membrane, cytoskeleton, organelles, and nuclei. We also discuss the heterogeneity, dynamics, and plasticity of SnCs' phenotype, including the SASP, and pro‐survival mechanisms. We advance on the multiple layers of phenotypic heterogeneity of SnCs, such as the heterogeneity between inducers, tissues and within a population of SnCs, discussing the relevance of these aspects to human health and disease. We also raise the main challenges as well alternatives to overcome them. Ultimately, we present open questions and perspectives in understanding the phenotype of SnCs from the perspective of basic and applied questions.

## Graphical abstract

This review describes the main subcellular alterations of senescent cells including features of plasma membrane, cytoskeleton, organelles, and nuclei. We also discuss the multiple layers of heterogeneity present in the biology of these cells as well as its dynamics and plasticity. At the end, we raise the main challenges in the field, as well open questions and perspectives in the area.

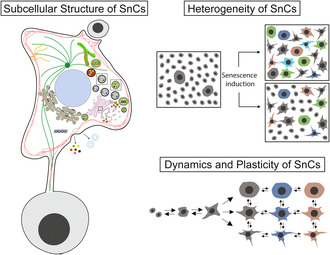

**Abbreviations**
CAFs: cancer‐associated fibroblasts
CCFs: cytoplasmic chromatin fragments
EVs: extracellular vesicles
PE: phosphatidylethanolamine
PS: phosphatidylserine
RS: replicative senescence
SH: Senescence heterogeneity
SAHF: senescence‐associated heterochromatin foci
SASP: senescence‐associated secretory phenotype
SCAPs: senescent cell anti‐apoptotic pathways
SnCs: senescent cells
SSC: single SnC
SEP: SnC‐enriched population
UPR: unfolded protein response

## 1 BASIC CONCEPTS IN CELLULAR SENESCENCE

**FIGURE 1:**
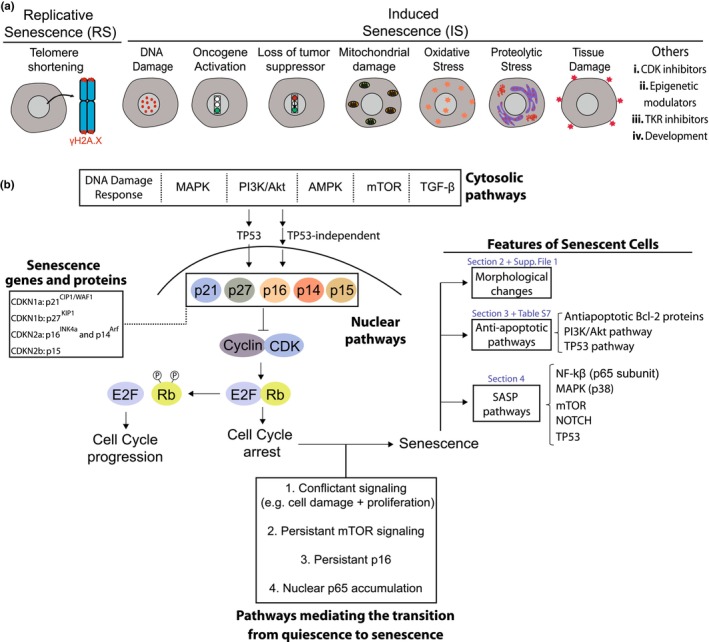
Overview of senescence inducers, molecular mechanisms, and cellular alterations. (a) Main senescence inducers, including replicative and induced (premature) senescence. (b) Top and center—cytosolic and nuclear pathways leading to cell cycle arrest. On the left, a box is shown with the genes and proteins that primarily carry out senescence. On the bottom are shown pathways driving in the progression from quiescence to senescence. The key features of SnCs are shown on the right, indicating specific sections of the manuscript where each is discussed.

Cellular senescence refers to a state of permanent growth arrest cells enters in response to intrinsic or extrinsic stimuli. The term, which comes from the Latin *senescentem*, that is, “to grow old,” was attributed to Hayflick and Moorhead (1961), who noticed that fibroblasts stopped replicating in vitro, reaching replicative senescence (RS) due to telomere shortening (Harley et al., 1990). In the following decades, inducers of premature senescence have been described, like DNA damage (DDIS), oncogene activation (OIS), loss of tumor suppressors, among others (Figure 1a). Looking at the big picture, SnCs can play beneficial or detrimental roles in human health and disease. Physiological responses like embryo development, wound healing, and tissue repair show a transitory increase in SnCs (Antelo‐Iglesias et al., 2021; Muñoz‐Espín et al., 2013; Storer et al., 2013). Conversely, the accumulation or dysfunction of senescent cells (SnCs) has been linked to the progression of diseases and the decline of tissue function in age‐related conditions (Adams et al., 2020; Gasek et al., 2021; He & Sharpless, 2017; Kirkland & Tchkonia, 2020; Koenig et al., 2022). Senescence also acts an endogenous anti‐cancer mechanism by blocking tumor initiation. Conversely, SnCs may contribute to tumor progression through the senescence‐associated secretory phenotype (SASP), a secretory program including soluble molecules and extracellular vesicles (Schmitt et al., 2022). Generally, the impact of SnCs on human physiopathology may hinge on factors such as the specific senescence inducer, the duration SnCs persist in the tissue, their phenotype, and the tissue in which the senescence occurs (Demaria et al., 2014; Paramos‐de‐Carvalho et al., 2021; Ritschka et al., 2017).

SnCs have several phenotypical and metabolic hallmarks (File S1; Gorgoulis et al., 2019). Despite decades of research, however, critical aspects of SnCs biology like subcellular structure, heterogeneity, and dynamics have only recently started to be comprehended. A holistic understanding of these features may contribute to a better grasp of the biological roles and functioning of these cells, as well as facilitate the identification of new markers and therapeutic strategies for promoting healthy aging. In this article, we provide a review of the primary subcellular characteristics of SnCs and explore the cause–consequence relationships underlying alterations in their behavior. We also delve into recent data on the heterogeneity, plasticity, and dynamics of SnCs, encompassing the SASP and the mechanisms that ensure SnCs' survival. At the end, we highlight the challenges and open questions in the field.

## 2 SUBCELLULAR CHANGES IN SNCS

**FIGURE 2:**
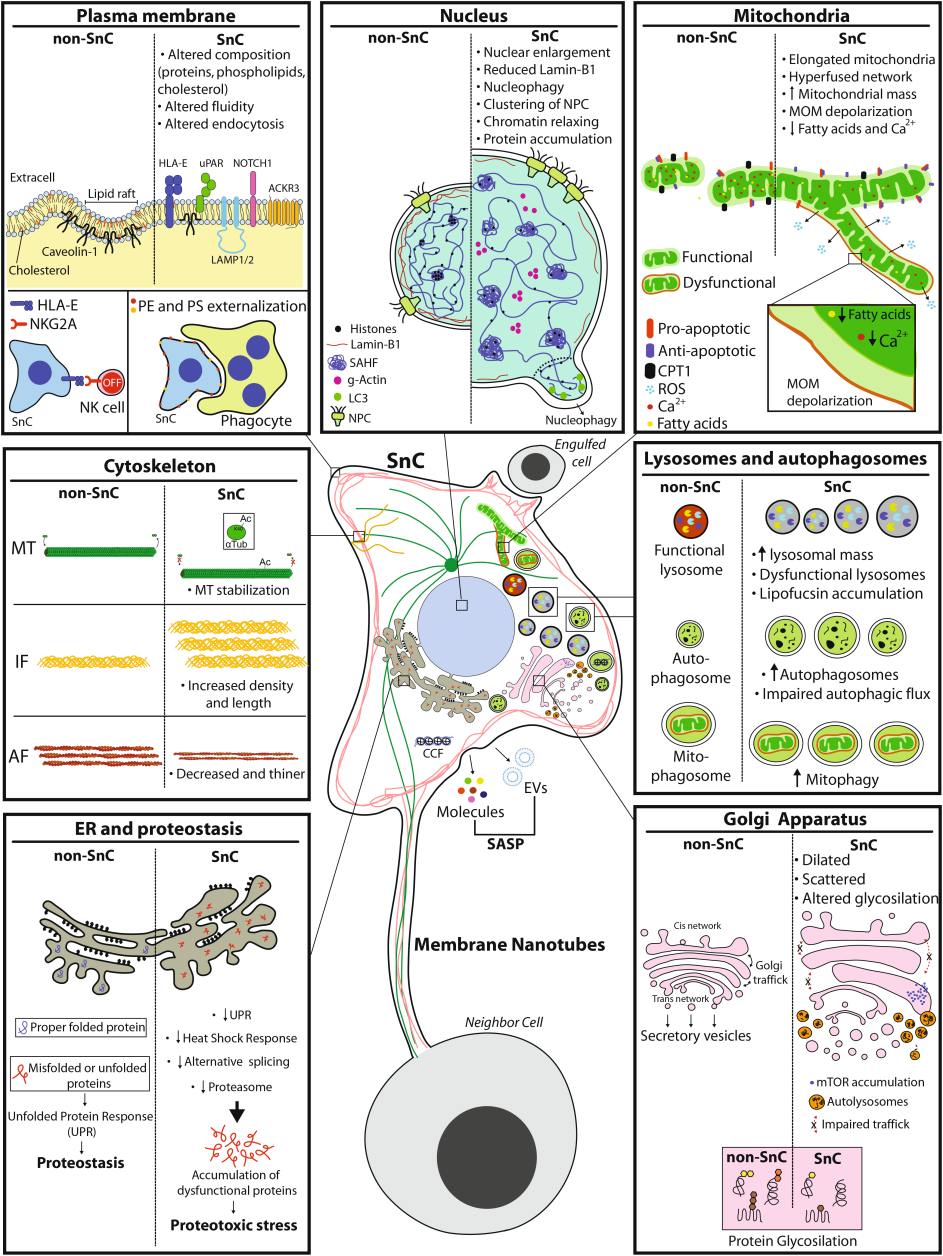
Subcellular alterations of SnCs. In the center is shown a representative model of a SnC with subcellular alterations. The boxes surrounding the cell represent details of the main subcellular changes comparing SnCs to non‐SnCs. Each box summarizes the main structural and functional changes observed in these components in SnCs. A detailed description of the subcellular changes, including the senescence inducer, cell model, and other findings, can be found in Tables [Link], [Link]. PE, phosphatidylethanolamine; PS, phosphatidylserine; SAHF, senescent‐associated heterochromatin foci; NPC, nuclear pore complex; MOM, mitochondrial outer membrane; MT, microtubules; IF, intermediate filaments; AF, Actin filaments or microfilaments; EVs, extracellular vesicles; CCF, chromatin; Ac, acetylation; ER, endoplasmic reticulum; UPR, unfolded protein response.

Unlike morphological or biochemical changes (File S1), the subcellular characteristics of SnCs exhibit greater complexity. Here, we built a SnC model (Figure 2) and tables (Tables [Link], [Link]) describing those features. Supplementary tables also have the information on senescence inducer and whether data are from SnC‐enriched population (SEP) or single SnC (SSC), which is relevant considering the heterogeneity of SnCs (discussed in Section 3), and the limitations that SEP data bring to the field (raised in Section 5). It is worth noting that even in studies with SSC evidence (e.g., microscopy, immunocytochemistry, or flow cytometry), results and discussion were mainly based on population (mean) data. It is also crucial to be aware that our goal is to describe subcellular characteristics found in cells that have *already acquired* a senescent phenotype, not alterations that *lead* to senescence.

### 2.1 Plasma membrane (PM)

The PM of SnCs shows expressive modification in its structure, composition, and functioning (Figure 2; Table S1). Initial evidence came from aged erythrocytes, which present increased phosphatidylethanolamine (PE) and phosphatidylserine (PS) exposure in the PM outer layer, which contributes to macrophages recognition (Boas et al., 1998; Larson et al., 2017). In fibroblasts under RS, there is a decrease in cholesterol levels and lipid rafts, leading to increased PM fluidity (Wi et al., 2021). PM changes are also related to membrane trafficking, including a more diffuse pattern of caveolin distribution (Wheaton et al., 2001), which may underlie the alterations observed in the endocytic capacity of SnCs (Shin et al., 2021). Finally, the PM proteome is altered in SnCs, mainly considering molecules involved in cell–cell contact or immune recognition. For instance, irradiation‐induced SnCs increase HLA‐E expression, which suppresses the activity of CD8+ T lymphocytes and NK cells (Pereira et al., 2019). Likewise, ACKR3, a chemokine ligand CXCL12 receptor involved in inflammation and the acquisition of senescent‐like profile in cancer‐associated fibroblasts (CAFs), is also increased in the PM of RS fibroblasts (Takaya et al., 2022). Together, this evidence suggests that changes in PM should affect not only the functioning of SnC but also responses to environmental factors and interactions with other cells, such as cells of the immune system.

### 2.2 Cytoskeleton

SnCs present alterations in cytoskeleton composition, structure, and/or functioning, leading to typical changes in their morphology and behavior (Figure 2; Table S2). Fibroblasts under RS increase the amount, density, and length of vimentin filaments, which may confer increased resistance to mechanical stress. On the other hand, actin filaments are decreased and thinner (Nishio et al., 2001; Nishio & Inoue, 2005), which may affect their migratory capacity. However, in contrast to the RS fibroblasts, DDIS in lung tumor cells reduce vimentin and change intermediate filaments organization and its structure due to increased HtrA2 serine protease (Hammer et al., 2022), suggesting that cytoskeleton alterations vary according to senescence inducers and/or cell models. About microtubules, OIS in epithelial cells reduces ROCK1 and histone deacetylase HDAC6, while increases α‐tubulin K40 acetylation (Moujaber et al., 2019). All these alterations result in microtubule stabilization, which may affect intracellular trafficking of vesicles and organelles. Finally, actin and tubulin are also crucial to establishing direct intercellular communication by SnCs through the formation of tunneling nanotubes. This process seems to be dependent on mTOR and CDC42, and allows SnCs to exchange intracellular components with neighboring cells, such as lysosomes, mitochondria, and proteins (Biran et al., 2015; Walters & Cox, 2021; Yasuda et al., 2011).

### 2.3 Mitochondria

Mitochondria dysregulation plays a role in the establishment of SnCs (Correia‐Melo et al., 2016; Farfariello et al., 2022; Wiley et al., 2016; Yu et al., 2020). However, the mitochondrial structure and functioning *in* SnCs are far more complex (Figure 2; Table S3). In general, SnCs show increased mitochondrial mass, associated with the overexpression of mitochondrial biogenesis proteins like PGC‐1α and TFAM proteins (Lee et al., 2002; Martínez et al., 2019). SnCs also show elongated and enlarged mitochondria, overexpression of MFN1 and PGAM5 proteins, which mediate mitochondrial fusion (Martínez et al., 2019; Yu et al., 2020). Interestingly, this phenotype may favor apoptosis resistance, thus contributing to senescence maintenance (Martini & Passos, 2023; Yu et al., 2020). Structurally, changes in cytoplasm‐to‐mitochondria trafficking may impair mitochondrial functions. For instance, CPT1‐B, an enzyme responsible for transporting fatty acids from the cytoplasm to the mitochondrial matrix, is reduced in the SnCs and impairs mitochondrial function (Wang et al., 2018). A similar perturbation was found for calcium transportation, leading to reduced levels in the intramitochondrial space with consequent mitochondrial depolarization and increased ROS production (Farfariello et al., 2022; Martínez et al., 2019). As alternatives to deal with impaired mitochondria, SnCs increase mitochondria turnover through mitophagy (Korolchuk et al., 2017; Yu et al., 2020), or change mitochondria particles by tunneling nanotubes with other cells (Walters & Cox, 2021). Finally, mitochondrial changes may also contribute to the SASP content. Mitochondria are an important source of damage‐associated molecular patterns like *N*‐formyl peptide, extracellular ATP, mtDNA, and cardiolipin. These molecules can be released from mitochondria to the cytoplasm—where they activate pro‐inflammatory pathways, and to the extracellular environment, where they recruit immune cells (Vizioli et al., 2020; Zlotorynski, 2020). It is important to mention that the cause‐and‐effect relationship of some mitochondrial alterations is still unclear. Moreover, most evidence comes from SEP, with scarce data on individual cells, including monitoring of mitochondrial dynamics and the impact of mitochondrial changes on the outcome of SnCs. In vivo, evidence from zebrafish telomerase mutants shows increased fragmentation of the mitochondrial network and reduced levels of ATP and SOD2 protein in gut ridges and testis tissue from old fish compared to young animals (El Maï et al., 2020). Mitochondrial fragmentation contrasts with in vitro data showing hyperfusion events, although it corroborates with dysfunction of the mitochondrial network. Notwithstanding, these differences reinforce the importance of developing new models or protocols to assess the subcellular structure and dynamics in vivo.

### 2.4 Lysosomes and autophagy

Lysosomes are one of the most studied organelles in SnCs (Figure 2; Table S4), as increased activity of lysosomal enzymes and the accumulation of lipofuscin are hallmarks of cellular senescence (Gorgoulis et al., 2019). In general, lysosome acidification and, consequently, their activity are hampered in SnCs (Colacurcio & Nixon, 2016; Curnock et al., 2023). Appropriately, SnCs might increase the number and size of lysosomes as a compensatory mechanism (Lee et al., 2006). Furthermore, lysosomal secretion is elevated in SnCs, with increased exposure of LAMP‐1/LAMP‐2 in the PM (Rovira et al., 2022). This process contributes to the secretion of relevant SASP molecules like chemokine ligands CCL2, CCL3, and CXCL12, in addition to cathepsin and SERPIN‐1. Finally, SnCs acquire the capacity to engulf neighboring cells and degrade them by lysosomal activity, a process that may contribute to SnC survival (Tonnessen‐Murray et al., 2019).

Lysosomes are also fundamental to autophagy. Some studies suggest that SnCs increase autophagy to compensate lysosome dysfunction, contributing to the turnover of membranes, organelles, and proteins (Gerland et al., 2003; Groth‐Pedersen et al., 2007; Rovira et al., 2022). In contrast, other studies show a decrease in autophagic activity in SnCs due to lysosomal defects like membrane permeabilization and reduced proteolytic capacity (Curnock et al., 2023; Ott et al., 2016). This reduction may hinder the degradation of defective large cellular components, such as mitochondria (Korolchuk et al., 2017) and chromatin (Han et al., 2020; Ivanov et al., 2013). Indeed, SnCs present cytoplasmic chromatin fragments (CCFs), small vesicles bounded by nuclear membrane that bud from the nucleus, carrying histones and DNA fragments (Dou et al., 2015; Han et al., 2020; Ivanov et al., 2013). This budding process is mediated by the interaction of nuclear LC3 with Lamin B1, leading to the reduction of the former (Dou et al., 2015), another hallmark of SnCs.

### 2.5 Nucleus

Nuclear enlargement is one of the most notable features of SnCs (Belhadj et al., 2023; Filippi‐Chiela et al., 2012; Heckenbach et al., 2022). Notwithstanding, changes in nuclear composition and functioning are also found (Figure 2; Table S5). SnCs present nuclear dysmorphism (i.e., alterations in nuclear shape) concomitant with the clustering of nuclear pore complex (Röhrl et al., 2021). This occurs in parallel with a decrease or loss of Lamin B1, an intranuclear filamentous protein essential to nuclear structure and functioning by anchoring the chromatin. This decrease seems to occur before other senescence molecular markers, suggesting Lamin B1 reduction as a potential earlier marker of senescence (Wang et al., 2017). These structural modifications occur in parallel with a global chromatin reorganization (Chandra et al., 2015; Shah et al., 2013), leading to the emergence of senescence‐associated heterochromatin foci (SAHF) due to the loss of interactions between chromatin and the nuclear lamina (Chandra et al., 2015). In addition to structural changes, SnCs also alter nuclear functioning. For instance, SnCs present a decrease in nucleocytoplasmic trafficking (Salunkhe et al., 2021), leading to the intranuclear accumulation of proteins, like globular actin (Kwak et al., 2004). However, as for changes in other subcellular compartments, the cause–consequence of nuclear alterations in SnCs still needs to be clarified, since nuclear alterations can also to be a driver to *induce* senescence (Park et al., 2021).

### 2.6 Other compartments

SnCs also have changes in other compartments, mainly involved in proteostasis (Figure 2; Table S6). It includes the enlargement of endoplasmic reticulum with significant declines in unfolded protein response (UPR) and heat shock response, leading to the accumulation of dysfunctional proteins (Sabath et al., 2020). Additionally, SnCs have dispersed and enlarged Golgi apparatus, which may underlie the presence of dysfunctional glycosylation and impaired Golgi trafficking observed in SnCs (Cho et al., 2011; Despres et al., 2019; Narita et al., 2011). Molecularly, Golgi's dispersion involves vacuolar ATPase *ATP6V0A2* downregulation, which is involved in protein glycosylation (Udono et al., 2015). Furthermore, the accumulation of mTOR and autolysosomes in the trans side of the Golgi apparatus may contribute to SASP synthesis and secretion (Mytych et al., 2020; Narita et al., 2011).

In conclusion, a large body of evidence demonstrates modification in the structure and composition of subcellular compartments in SnCs, which depend on the cell type and the senescence‐inducing stimulus. Some alterations impact intracellular functioning and the behavior of SnCs, including non‐autonomous effects and SASP production. However, several cause–consequence relationships still need to be discovered. Furthermore, many conclusions were based on populational data. Notwithstanding, as can be seen in Tables [Link], [Link], many studies have evidence from single cells, like flow cytometry or microscopy, but analyses of variability or heterogeneity between individual SnCs were not carried out. Even scarcer are multiple subcellular changes in individual SnCs and evidence from the dynamics of subcellular compartments, which is crucial for understanding the behavior and fate of SnCs.

## 3 HETEROGENEITIES, PLASTICITY, AND DYNAMICS OF CELLULAR SENESCENCE

### 3.1 Phenotypic heterogeneities of SnCs

Senescence heterogeneity (SH) encompasses multiple layers, from differences within SnCs of cell populations (intra‐popSH) to variances between SnCs triggered by different inducers (inter‐inducer SH) or originating from diverse tissues (inter‐tissue SH). In this section, we discuss the primary evidence and potential clinical implications related to different layers of SH.

The intrapopulational heterogeneity of SnCs (intra‐popSH) was initially shown in aged fibroblasts. Based on their transcriptomes, five main clusters of cells were found: one proliferative, two quiescent, and two senescent (Hernandez‐Segura et al., 2017). Confirming this finding, fibroblasts under RS showed two main clusters of SnCs, one enriched with the expression of cell cycle genes and the other with SASP genes (Wiley et al., 2017). After that, further studies confirmed the presence of different transcriptomes in populations of other cell types like mesenchymal cells under RS (Taherian Fard et al., 2023) and aging melanocytes (Victorelli et al., 2019). For pancreatic β cells, levels of p16 vary significantly within and between β cells islets of a same individual (Aguayo‐Mazzucato et al., 2017). This is clinically relevant since senescent β cells, which increase in type‐2 diabetes mellitus patients (Aguayo‐Mazzucato et al., 2019), decrease insulin secretion and increase pro‐senescent SASP (Aguayo‐Mazzucato et al., 2019; Sone & Kagawa, 2005). Not surprisingly, intra‐popSH also occurs in neoplastic cells like cancer cells (Neurohr et al., 2019; Saul & Kosinsky, 2021; Troiani et al., 2022) and melanocytes from skin naevi (Michaloglou et al., 2005; Victorelli et al., 2019). Potential players mediating the transcriptional heterogeneity of SnCs include the AP‐1 transcription factor (Martínez‐Zamudio et al., 2020) and the H3‐specific demethylase KDM4 (Zhang et al., 2021). Likewise, Notch 1 signaling fluctuates and controls the variation of SASP composition in fibroblasts (Hoare et al., 2016). Interestingly, the heterogeneity in mitochondrial function and ROS production seems to be directly involved in the intra‐popSH observed in fibroblasts under RS (Passos et al., 2007).

Different senescence‐inducing factors can also lead to SnCs with varying transcriptomes (Hernandez‐Segura et al., 2017; Tang et al., 2019; Wechter et al., 2023), characterizing the inter‐inducer SH. After irradiation‐induced DNA damage, for instance, a single cluster of SnCs considering their transcriptome was observed (Tang et al., 2019), while two subpopulations of SnCs were found in fibroblasts undergoing senescence induced by replicative stress or etoposide‐induced DNA damage (Tang et al., 2019; Wechter et al., 2023; Wiley et al., 2017). Indeed, the degree of heterogeneity seems to depend on the senescence‐inducing stress, since the transcriptomes of cells induced to senescence by different damage‐inducing agents, although different, are more similar among them compared to RS (Wechter et al., 2023). Different senescence inducers can also lead the same cell type to produce a SASP with different compositions and functions. For example, while in the healing process, the SASP produced by SnCs contributes to cell proliferation and tissue repair (Huang et al., 2022), the SASP produced by melanocytes in RS represses aged skin regeneration (Victorelli et al., 2019). Finally, differences in gene expression triggered by various senescence inducers can also underlie the variations observed in the pattern of senescence markers. For instance, K‐Ras^G12V^‐induced senescence is associated with the appearance of SAHF with cellular and nuclear enlargement (Pantazi et al., 2019). In contrast, cRAF‐induced senescence leads to SAHF formation without nuclear enlargement (Jeanblanc et al., 2012).

The phenotype of SnCs from different tissues also exhibit differences, characterizing the inter‐tissue SH. The analysis of 2365 genes in senescent fibroblasts, keratinocytes, and melanocytes showed that only up to 5% of them were commonly expressed, which the authors named “Core Senescence‐Associated Signature” (Hernandez‐Segura et al., 2017; Saul et al., 2022). As a consequence, SnCs from different tissues exhibit variations in both the composition and function of the SASP, and in the clearance process (Hernandez‐Segura et al., 2017; Ritschka et al., 2017; Storer et al., 2013; Victorelli et al., 2019). This observation may have clinical implications since different cell types from the same organ can undergo senescence, such as in kidneys (where senescence has been noted in tubular epithelial cells, endothelial cells, mesangial cells, and podocytes; Huang et al., 2022), liver (with reported senescence in hepatocytes, stellate cells, and cholangiocytes; Aravinthan & Alexander, 2016), lungs (with observed senescence in fibroblasts, epithelial cells, and endothelial cells) (Hansel et al., 2020), and brain (in which both glia and neurons can undergo senescence; Rachmian & Krizhanovsky, 2023). Therefore, given that the phenotype of SnCs from different tissues can differ within the same organ, a significant challenge in the field is to mitigate the detrimental effects of SnCs while preserving their beneficial roles.

Nevertheless, although the transient enrichment of SnCs can play helpful roles in physiological processes like wound healing (Demaria et al., 2014) and embryonic development (Storer et al., 2013), their accumulation in aging and aging‐associated diseases contributes to loss of tissue function (Baker et al., 2011; Chang et al., 2016; Ogrodnik et al., 2021). The enrichment and prolonged survival of SnCs in that contexts are facilitated by the upregulation of pro‐survival signals and the simultaneous blocking of cell death pathways (Zhu et al., 2015), a feature collectively referred to as senescent cell anti‐apoptotic pathways (SCAPs; Table S7). The main SCAPs are Bcl‐2 and TP53/p21 pathways (Fuhrmann‐Stroissnigg et al., 2017; Zhu et al., 2016, 2017). Other pathways contributing to SnC survival are ephrins/tyrosine kinase (Hafner et al., 2005; Ståhl et al., 2013), HIF‐1α (Alique et al., 2019; Welford et al., 2006), and HSP90 (Fuhrmann‐Stroissnigg et al., 2017). Despite the primary evidence on SCAPs coming from population studies (see Table S7), initial evidence shows that SCAPs are also heterogeneous among SnCs from the same population (Deryabin et al., 2021; Troiani et al., 2022; Zhang, Pitcher, et al., 2023; Zhang, Zheng, et al., 2023). This heterogeneity may underlie the different outcomes in response to senolytics, whereas some SnCs undergo senolysis and others survive, (Selt et al., 2023; Zhu et al., 2017), another evidence supporting the intra‐popSH.

**FIGURE 3:**
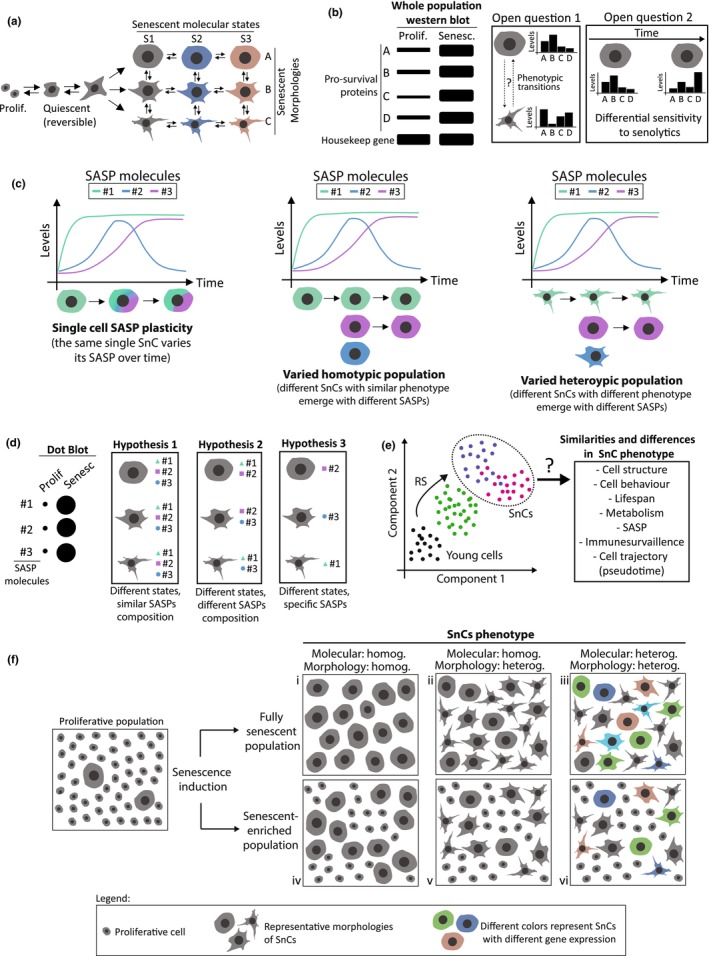
Open questions in cellular senescence. (a) Phenotypic plasticity of SnCs, including molecular states (S1–S3) and morphologies (a–c). (b) Heterogeneity and plasticity of SCAPs. *Left*—schematic western blot representing the increase in the expression of pro‐survival proteins in SnCs. *Open question 1*: Do transitions between different states or phenotypes lead to changes in SCAPs? *Open question 2*: Do SCAPs vary over time in single SnCs? (c) Dynamics of SASP over time. #1, #2, and #3 represent SASP molecules. (d) Heterogeneity and plasticity of SASP. *Left*—schematic dot plot representing the increase in SASP in a senescence‐enriched population. (e) Representative model of dimensionality reduction to identify SnCs' subpopulations along the progression of the phenotype. RS, replicative senescence. (f) In each condition from *i to vi*, intra‐populational heterogeneities are shown: Colors represent different patterns of gene expression, while different cell shapes represent morphometric heterogeneity.

Furthermore, although different senescence‐inducing stresses may act by similar SCAPs, the effector proteins responsible for cell survival may vary in response to different inducers (Yosef et al., 2016), depending on the genetic background and the intensity of cellular damage (Alsayegh et al., 2015; Baar et al., 2017; Yosef et al., 2017). Taking this into account, the combination of senolytics may be more efficient in eliminating heterogeneous populations of SnCs than single treatments (Storer et al., 2013; Zhu et al., 2017), which needs to be confirmed by clinical trials. In addition, the specific role played by the different cell types that undergo senescence or SnCs living in different phenotypic states (Figure 3a) remains to be elucidated. We also do not know if all SnCs from a given population express similar SCAPs (Figure 3b—open question 1) or whether individual SnCs change the expression of SCAPs during their lives (Figure 3b—open question 2).

### 3.2 Dynamics and plasticity of SnCs

Adding complexity to the biology of SnCs, they also vary their transcriptome over time, suggesting a dynamic state (González‐Gualda et al., 2021; Hernandez‐Segura et al., 2017). The intra‐popSH increases from Days 0 to 10 after DNA damage induced by etoposide, leading to two different senescence programs, one characterized by classical senescence markers like p16, and the other by RNA splicing and long non‐coding RNAs (Wechter et al., 2023). Likewise, during RS or DDIS, cells display a greater degree of variability in their transcriptome compared to the quiescent state (Hernandez‐Segura et al., 2017; Wiley et al., 2017). Evidence of such transcriptomic dynamics is highly relevant as there remains a need for a deeper understanding of the mechanisms underlying these state transitions. Currently, scRNA‐seq has shed some light on this issue. From around 7700 genes, 1311 were differentially expressed between senescent and quiescent fibroblasts regardless of senescence inducer (Hernandez‐Segura et al., 2017), a proportion corroborated by another study using fibroblasts under RS (Tang et al., 2019). Intracellular pathways mediating the transition from quiescence to senescence are shown in Figure 1b. Directly or indirectly, these pathways modulate the activity of Rb‐E2F complex, thus controlling the cell fate (Fujimaki & Yao, 2020). Other players controlling the cell decision between proliferation, quiescence, or senescence are p21 levels (Hsu et al., 2019) and proliferation status (i.e., if the cell is cycling or not) when senescence initiates (Wechter et al., 2023).

Despite the growing body of evidence suggesting different layers of phenotypic heterogeneity and dynamics in SnCs, it is necessary to add supporting data considering the spatiotemporal dynamics of senescence markers in vivo. Initial evidence shows that in mice, levels of p16 were dynamic during embryonic and postnatal development, with p16 increasing sharply during aging in the heart, brain, kidney, and liver. A robust postnatal increase was also observed for SASP molecules such as IL‐6, TGF‐1β, VEGFA, and MMP‐9. In contrast, despite also dynamic, p21 and p19 increased only moderately in old animals. Interestingly, although variable expression levels of these genes varied in different organs during aging, the spatiotemporal heterogeneity between organs is attenuated in old animals (Safwan‐Zaiter et al., 2022). Besides aging, a recent study evaluating the expression of senescence markers in neurodevelopmental defects induced by valproate found no change in the expression of p16 and p21 but observed an increase of p19 over time (Rhinn et al., 2022). Finally, other age‐associated diseases with dynamic variations in senescence markers throughout pathogenesis are metabolic diseases. In mice, excessive caloric intake induces a gradual increase of senescent pancreatic β cells (Sone & Kagawa, 2005), while the progressive senescence of preadipocytes may also contribute to insulin resistance (Gao et al., 2020). In hypercaloric obesogenic models, adipocytes increase p21 in the early stages, followed by an increment of p16 in the later stages (Wang et al., 2022). This observation is translationally relevant since type 2 diabetes mellitus itself increase SnCs within the adipose tissue in mice (Ahima, 2009), while senescence rise the risk of T2DM development and worsening (Burton & Faragher, 2018), thus creating a progressive positive feedback loop.

In addition to cell cycle inhibitors, other markers such as DNA damage indicators (e.g., histone H2A.X foci) also demonstrate an increase throughout the aging of mice in several organs, such as lung, spleen, liver, small intestine, and testis (Wang et al., 2009). Moreover, there is a significant increase in the activity of SA‐β‐gal in the skin of elderly individuals compared to young individuals in humans (Dimri et al., 1995), mice (Wang et al., 2009), and zebrafish (Kishi et al., 2003). Likewise, the activity of SA‐β‐gal increased as a function of time in T lymphocytes in humans (Martínez‐Zamudio et al., 2021) and in the hippocampus of old animals compared to young ones (Geng et al., 2010).

It is worth mentioning that we did not consider variations in the phenotype of individual SnCs over time as a type of heterogeneity, but rather cellular plasticity, one of the least understood aspects of SnCs. Little or nothing is known regarding differential changes in cellular and subcellular structures of SnCs, either as a cause or a consequence of the quiescence‐to‐senescence transition, nor if the same SnCs can assume different phenotypic states over time (Cohn et al., 2023; Figure 3a). Although gene expression changes in the population over time after senescence induction (Ge et al., 2022; Hernandez‐Segura et al., 2017), it is not possible to affirm that the same cell alters their transcriptome, which could also lead to dynamic changes in SCAPs mechanisms (Figure 3b—open question 2) or SASP composition (Figure 3c). Therefore, in addition to transcriptomics, it is necessary to study structural heterogeneity and dynamics by investigating cellular and subcellular structure, behavior, and fate of single SnCs, allowing the full characterization of basic phenotypic aspects of SnCs.

## 4 SASP: TYPES AND HETEROGENEITY

SASP comprises soluble factors and extracellular vesicles secreted by SnCs that can modulate local or systemic responses (Acosta et al., 2013; Budamagunta et al., 2023; Coppe et al., 2010). SASP can be highly heterogeneous depending on the SnC type and the senescence inducer (Basisty et al., 2020). Functionally, it can be classified into proapoptotic, pro‐fibrotic, and pro‐inflammatory, which have different roles and effector molecules (Chaib et al., 2022; Coppe et al., 2010; Tchkonia et al., 2013).

IL‐6 and TGF‐β seem to be the most relevant molecules of pro‐fibrotic SASP, affecting matrix deposition and mesenchymal cells functioning (Barron et al., 2010; Liu et al., 2022). IL‐6 is a pro‐inflammatory molecule mediating tissue fibrosis through the JAK–STAT pathway (Xu, Palmer, et al., 2015; Xu, Tchkonia, et al., 2015; Yasuda et al., 2021). TGFβ is also associated with RAS‐induced senescence, where Notch 1 drives its expression and promotes the release of pro‐inflammatory cytokines like IL‐1, IL‐6, and IL‐8 (Hoare et al., 2016). The proapoptotic SASP, in turn, induces tissue destruction through the secretion of apoptosis‐inducing molecules, to which SnCs themselves are resistant (Chaib et al., 2022). Consequently, it leads to loss of tissue function and secondary senescence, contributing with the progression of age‐related disorders (Covarrubias et al., 2020; Feng et al., 2023; Herdy et al., 2022; Moiseeva et al., 2023; Yao et al., 2021). The proapoptotic SASP added to the immunosuppressive properties of SnCs surpasses the immunological clearance capacity, resulting in the progressive accumulation of SnCs (Chaib et al., 2022). Finally, SnCs can produce a pro‐inflammatory SASP, which lead to a chronic and sterile inflammatory state known as inflammaging. NF‐kB is increasingly recognized as a primary regulator of pro‐inflammatory SASPs (Bigot et al., 2012; Mi et al., 2022; Nizamutdinova et al., 2016; Schlett et al., 2023), as discussed below. The inflammaging, commonly observed in aging and chronic diseases, impairs the function and the regenerative capacity of non‐SnCs, while promoting or accelerating disorders like brain insulin resistance (Barone et al., 2021; Haas et al., 2020), tumorigenesis (Kuilman et al., 2008; Yasuda et al., 2021), muscle wasting (Huang et al., 2023; Moiseeva et al., 2023), and cognitive decline (Budamagunta et al., 2023; Herdy et al., 2022; Ogrodnik et al., 2021). Functionally, SASP classification into these three profiles is due to the predominant but not exclusive effect it exerts, since numerous molecules may be common to all SASPs. Thus, it is possible that a secretory phenotype plays a pro‐fibrotic effect while also promoting tissue inflammation (Feng et al., 2023; Gopinath & Rando, 2008; Yao et al., 2021). Finally, for heterogeneous populations of SnCs, it is plausible to assume that SASPs with widely varying constitutions are produced.

The SASP also contains extracellular vesicles (EVs), the effects of which depend on cell source and the senescence inducer (Wallis et al., 2020). During OIS, SnC‐derived EVs contribute to the paracrine senescence in neighboring fibroblasts through the modulation of IFN pathway (Borghesan et al., 2019). In contrast, EVs released by SnCs of the tumor microenvironment promote the proliferation of cancer cells through the activation EphA2/ephrin‐A1 signaling (Takasugi et al., 2017). EVs released by SnCs also contain cell‐free DNA, including mtDNA, which activates immune responses, resulting in the release of inflammatory factors (Iske et al., 2020). Therefore, SnC‐derived EVs have the potential for the identification of new markers in senescence, screening of SnCs, and studies of the heterogeneity and dynamics of senescence.

It is worth noting that some authors suggest classifying the SASP based on the senescence type or the senescence inducer (Basisty et al., 2020; Oguma et al., 2023), which is relevant since there are significant differences in the secretome depending of that variables (Figure S1). Furthermore, the SASP is not a static entity but varies over time, at least in vitro (Basisty et al., 2020). Whether this dynamic also occurs in vivo, however, remains open. Furthermore, it is not known whether the same cells change their SASP over time or whether there is a transient enrichment of different SnC phenotypes over time (Figure 3c). Regardless of the classification, it is essential to keep in mind that SASP can have beneficial or harmful effects on human health, depending on factors like its composition, the cell type, and for how long it persists in the tissue.

### 4.1 Molecular mechanisms controlling the SASP

The SASP is governed by various transcriptional and epigenetic mechanisms. Main signaling pathways controlling SASP are PI3K/Akt/mTOR, p38MAPK, and TP53. mTOR increases SASP through the upregulation of MAPKAPK2 (Herranz et al., 2015; Kucheryavenko et al., 2019). In cancer, mTOR‐mediated IL‐1α production by SnCs contributes to tumor progression (Laberge et al., 2015). Another pathway promoting the SASP is p38MAPK, whose overexpression per se increased SASP, independently of DNA damage (Freund et al., 2011). For mTOR and p38MAPK pathways, the main effector molecule is NF‐κB, a transcription factor playing crucial role in producing pro‐inflammatory molecules. Metformin, an antidiabetic drug, inhibits NF‐κB activation and selectively represses SASP genes (Moiseeva et al., 2013). This is clinically relevant since molecules secreted by senescent pancreatic β cells, like CXCL1, IL‐6, and TNFα may contribute to the progression of type 2 diabetes (Aguayo‐Mazzucato et al., 2019; Xu et al., 2003; Xu, Palmer, et al., 2015; Xu, Tchkonia, et al., 2015; Zaragosi et al., 2010). Other transcription factors involved with the expression of SASP molecules are C/EBPs family and TP53 (Atwood & Sealy, 2011; Sheekey & Narita, 2023; Wallis et al., 2020). TP53 negatively regulates SASP, so its absence, which occurs in around 50% of human cancers, and enhances pro‐tumoral SASP. Nutlin, a MDM2 inhibitor, increases TP53 activity, attenuating the expression of pro‐inflammatory cytokines like IL‐6 and IL‐1α (Pandya et al., 2022) Finally, NLRP3 modulate the SASP of OIS through a non‐canonical pathway involving caspase‐5 (Wiggins & Clarke, 2019).

The observation that numerous mechanisms contribute to the SASP greatly underscores its heterogeneity and dynamics. Adding even more complexity, transcriptional pathways modulating SASP are interconnected. The tumor suppressor SOCS‐1, for instance, activates TP53 and inhibits NF‐κB, leading to a distinct SASP associated with tumor suppression (Calabrese et al., 2009). In TNF‐α‐induced senescence, NF‐κB crosstalks with JAK/STAT signaling to regulate the SASP (Kandhaya‐Pillai et al., 2017). Finally, in DDIS and OIS both p38 and mTOR can be activated by TP53, thus modulating the SASP in a complex manner (Sheekey & Narita, 2023). Overall, this evidence reflects on the intricate network of transcriptional pathways involved in SASP regulation, underscoring the need for further research to unravel the precise molecular mechanisms underlying this phenotype.

Considering epigenetics, two main mechanisms regulate the SASP: (a) DNA methylation and chromatin changes, and (b) the activation of the cGAS‐STING pathway. Although interconnected, these mechanisms can be independently induced. Persistent DNA damage triggers the degradation of G9a and GLP histone H3K9 dimethyl transferases via the proteasome, leading to a relaxed chromatin in SnCs that allows the production of SASP (Takahashi et al., 2012). Other epigenetic modifiers contributing to the maintenance of active SASP‐related loci are MLL1 and HMGB‐2 (Capell et al., 2016; Guerrero & Gil, 2016; Zhao et al., 2020). Furthermore, the activation of the ATM pathway promotes the binding of poly‐ADP‐ribose to macroH2A.1.1 by PARP‐1, causing the removal of macroH2A.1 and the consequent activation of SASP genes expression (Kozlowski & Ladurner, 2015; Ohanna et al., 2011).

In advanced stages of cellular senescence, intensified DNA damage response leads to the generation of cytoplasmic DNA fragments due to cytokinesis blockage and impaired nuclear division. These fragments activate DNA sensors, including the cGAS‐STING pathway, which plays a crucial role in innate immune responses (Schmitz et al., 2023; Yang et al., 2017). Retrotransposon elements, specifically LINE‐1, have been observed in SnCs, leading to cGAS‐STING pathway activation (Gamdzyk et al., 2020). Such accumulation has been associated with age‐related chronic inflammation and premature aging syndromes, where the inhibition of the retrotransposon replication attenuates chronic inflammation (Della Valle et al., 2022). Another epigenetic mechanism controlling the SASP is the reduction of Lamin B1, which destabilizes the nuclear structure leading to micronuclei formation, cGAS‐STING activation and IFN production (Mackenzie et al., 2017). Furthermore, during OIS, enhancer landscapes are remodeled, recruiting BRD4 to super‐enhancer elements adjacent to SASP genes (Thompson et al., 2019). Understanding the epigenetic regulation of SASP provides valuable insights into cellular senescence and age‐related conditions. Targeting specific epigenetic modifications and associated enzymes may hold therapeutic potential for modulating SASP, mitigating senescence, and improving tissue function.

The intricate cell signaling pathways and the intrinsic variability in their production define a prominent level of complexity associated with SASP production. Furthermore, unknown crosstalk between SASP pathways may occur, creating a complex scenario that needs to be clarified. The challenges lie in understanding: (a) SASP complexity using simplistic models that limits translational validations, (b) the dynamics of SASP in single cells (Figure 3c,e), (c) the SASP heterogeneity and plasticity (Figure 3d,e); and (d) the most effective way to modulate SASP without impacting non‐SnCs and normal physiology.

## 5 CURRENT CHALLENGES

The intricate nature of senescence coupled with technical issues and limited experimental models have hindered even more substantial progress in understanding the phenotype of SnCs, mainly considering in vivo data. In this section, we discuss the main challenges in the field, the issues that keep these challenges unsolved, and alternatives to overcome them.

Primary cell cultures and even cell lines exhibit varying degrees of heterogeneity. Moreover, tissues and organs comprise different cell types with diverse phenotypes. Given these distinct levels of biological organization, not all cells from a biological sample may simultaneously undergo senescence after exposure to a senescence‐inducing stimulus (Bourgeron et al., 2015; Kirschner et al., 2020; Taherian Fard et al., 2023; Figure 3f). The detection of varying levels of senescence markers in individual SnCs confirms the existence of intra‐popSH, suggesting (a) different senescent states, (b) differential sensitivity to senescence, or (c) diverse kinetics in achieving the senescent phenotype (Goy et al., 2023; Troiani et al., 2022; Wang et al., 2016). Due to the non‐binary progressive nature of senescence, not all SnCs are in the same phenotypic states at the time of analysis (Ashraf et al., 2023; Hernandez‐Segura et al., 2017; Sturmlechner et al., 2021). Furthermore, different cell types or subpopulations of SnCs can contribute to average levels of senescence markers in a population (Jeyapalan & Sedivy, 2013; Sturmlechner et al., 2022; Troiani et al., 2022), as well as non‐SnCs can also contribute with features attributed to SnCs (Gorgoulis et al., 2019), which is especially important in studies using primary cells from human or animal samples (Bahar et al., 2006; Delfarah et al., 2021; Tuttle et al., 2021). These variables are particularly significant because the majority of studies use senescence‐enriched cellular populations (SEP), rather than examining a population containing only SnCs (González‐Gualda et al., 2021; Jeyapalan & Sedivy, 2013; Liu et al., 2019; Menegotto et al., 2017; Silva et al., 2020) (Figure 3f). To overcome this barrier, techniques designed to isolate SnCs are essential, like cell sorting, scRNA‐seq, tissue microdissection, or immunocytochemistry (Bertolo et al., 2020; Gruber et al., 2010; Magkouta et al., 2023; Wallis et al., 2022; Wang et al., 2016).

Strategies for isolating single SnCs may also shed light on the longstanding concern regarding the sensitivity and specificity of senescence markers (Ashraf et al., 2023; Hall et al., 2017; Safwan‐Zaiter et al., 2022). Based on this concern, efforts have been made to define the necessary and sufficient set of features to characterize a SnC (Gorgoulis et al., 2019; Hernandez‐Segura et al., 2018), a need supported by the observation that two or more markers in the same SnC can improve the accuracy of the analysis (Ashraf et al., 2023; Baldasso‐Zanon et al., 2024). However, it is challenging to access multiple markers in the same SnC (González‐Gualda et al., 2021). Equally challenging is tracking the phenotypic dynamics of SnCs or the transition of individual cells from the proliferative to the senescent state (Begnini et al., 2022). Understanding this switch has the potential to reveal new mechanisms as well as new targets for senotherapies.

Likewise, among the most complex challenges within cellular senescence certainly are in vivo analyses. Most studies using primary samples or samples from in vivo models analyze the expression of molecular markers, in some cases in single cells as cited throughout the manuscript (Hernandez‐Segura et al., 2017; Michaloglou et al., 2005; Neurohr et al., 2019; Tang et al., 2019; Troiani et al., 2022; Victorelli et al., 2019; Wechter et al., 2023; Wiley et al., 2017) or the activity of senescence‐associated enzymes. As anticipated, in vivo samples are predominantly examined in aging and age‐related diseases, in which the most widely investigated hallmark of senescence is the increase of p16 (Burd et al., 2013; Krishnamurthy et al., 2004; Muss et al., 2020; Raffaele et al., 2020; Ressler et al., 2006; Tuttle et al., 2021). Furthermore, increased activity of SA‐β‐gal (Dimri et al., 1995; Doura et al., 2016; Kishi et al., 2003; Raffaele et al., 2020), loss of Lamin‐B1 (Freund et al., 2012; Matias et al., 2022; Wang et al., 2017), and alterations in nuclear morphometry (Freund et al., 2012; Matias et al., 2022; Pathak et al., 2021) are also observed in vivo. Nevertheless, contrary to molecular markers, many phenotypic hallmarks of cellular senescence easily assessed in vitro are complicated and even unfeasible to measure in vivo, like cell size and shape, or alterations in the structure and dynamics of subcellular compartments. These limitations are due to technical and biological issues, including loss of original tissue architecture when cells are isolated and cultured in vitro, the need to fix primary samples before analysis, and the difficulty of tracking single cells in live tissue. Furthermore, the varied nature of the senescent process and the inherent complexity of living organisms pose additional challenges. Therefore, transgenic mice expressing fluorescent reporters under the control of p16 (Demaria et al., 2014; Liu et al., 2019; Ogrodnik, 2021) or p21 promoters (Wang et al., 2021; Yi et al., 2023) added to other animal models like *Drosophila melanogaster* (Chen et al., 2014), *Caenorhabditis elegans* (Ezcurra et al., 2018; Lohr et al., 2019), or Zebrafish (*Danio rerio*) (Kishi et al., 2008; Morsli et al., 2023) may contribute significantly to generate in vivo evidence. Likewise, techniques and protocols for detecting and assessing senescence in preserved or live tissue are underway, including the isolation of SnCs, ex vivo analyses, new probes allowing in vivo imaging, supravital microscopy, and in vivo cell tracking (Biran et al., 2017; Rabinowitz & Cui, 2023). Together with molecular data, these strategies will contribute to understanding the structure and behavior of SnCs beyond revealing potential new targets for senotherapies.

## 6 OPEN QUESTIONS AND CONCLUSIONS

Despite significant advances in the characterization of SnCs, many questions about the biology of these cells remain open (Table 1). Firstly, it is necessary to understand which markers are necessary and sufficient to define that a cell is in a “full” or “deep” senescent state. Similarly, the dynamics and adaptability of SnCs still need to be better understood, including how plastic those cells are for the expression of SCAPs, the structure of intracellular compartments, or SASP composition, the pathways governing these transitions, and how intense these phenotypic variations must be to influence the non‐autonomous role played by SnCs. Understanding these aspects may also allow us to infer whether intra‐popSH occurs due to different states in different SnCs or the plasticity of individual SnCs. Finally, it is also imperative to comprehend the heterogeneity and the cause‐and‐effect between subcellular features and the outcome of SnCs. New evidence regarding the above questions can also contribute to understanding questions such as how long an SnC lives and whether death is the only possible outcome.

**TABLE 1:** Open questions about the phenotype, dynamics, and functions of senescent cells.

Topic	Open questions	Goals and perspectives
1. Senescence markers and phenotype	Is it possible to define a “full” and unique senescent phenotype? Which markers are necessary and sufficient for that?	Search for more specific and sensitive markers, and molecular signatures for SnCs
2. The plasticity and dynamics of SnCs	Can individual SnCs assume different cellular states? How plastic and dynamic are SnCs? What are the sequential molecular and subcellular changes until establishing the senescent phenotype? Is intra‐popSH due to different cells or the same cells changing their phenotype over time?	Investigate the dynamic of behavior and structure of SnCs and their compartments through live cell imaging Integrate multiple molecular and subcellular markers of different cellular states Investigate the drivers of SnCs' plasticity and the molecular trajectory of SnCs through pseudotime
3. Transition from quiescence to senescence	What are the mechanisms regulating the point‐of‐no‐return from quiescence to senescence? How can this be exploited to prevent or treat age‐related diseases?	Understand better the cause–consequence relationship between morphological and structural alterations of SnCs through live cell imaging and scRNA‐seq
4. Living as a SnC	How long does a SnC live? What factors or structural features affect the fate of a SnC? Are there differences in the phenotype and behavior of SnCs induced by different stressors? Is the only outcome for a SnC its death?	Improve equipment and protocols for the long‐term tracking of live SnCs, including new reporters and techniques for cell behavior analysis Develop new models to study senescence, especially in vivo
5. SnC survival and senotherapies	How heterogeneous and dynamic is the expression of SCAPs in SnCs? Could the function or structure of subcellular compartments from SnCs be used as targets for senotherapies?	Build engineered cell models to assess the expression of pro‐survival proteins or pathways in live cells Modulate the structure, dynamics, or proteins in subcellular compartments to sensitize SnCs
6. SASP and chronic conditions	How does SASP change their composition and biological role in chronic conditions? Is this pattern tissue‐specific? How do we define the most important SASP molecules for each condition?	Combine molecular biology and bioinformatics to identify regulatory players of SASP Create predictive spatial maps to explore heterogeneous populations of senescence in different conditions and contribute to developing new senomorphic approaches
7. Senescence activation for physiological roles	What are the triggers and drivers of senescence in physiological contexts (e.g., tissue repair and embryo development)?	Develop protocols and techniques for in vivo investigation Seek different markers involved in these processes and compare the phenotype of SnCs from different physiological contexts

*Note*: Seven hot topics are shown, with the main open questions, goals, and perspectives.

A better understanding of essential features of SnCs can also contribute to translational issues in which cellular senescence appears to be relevant. Questions like the role of SASP in acute responses and chronic conditions and the most relevant SASP molecules for pathophysiological responses may allow the mitigation of detrimental impacts or the increase of beneficial effects played by SnCs. It is also mandatory to uncover novel avenues for senotherapies, such as senolytics (for instance, by targeting the heterogeneity of this phenotype), senopreventives (by elucidating mechanisms allowing senescence entry), and senomorphics (by affecting the detrimental effects of SASP selectively). Nevertheless, several barriers need to be overcome to allow the clinical application of basic concepts in cellular senescence, such as the lack of specific therapies to reduce detrimental but not beneficial effects played by SnCs, the best time to affect senescence in pathophysiological responses, and how to assess the effectiveness of senotherapies.

In conclusion, although several clinical trials targeting SnCs are ongoing, various questions about the biology of SnCs remain open, resulting in a gap between molecular and cellular data. Concerning the need, initiatives aiming to create openly accessible atlases of SnCs should contribute enormously to the area (SenNet Consortium, 2022). Advances in understanding the subcellular structure, the heterogeneity, and the dynamics of SnCs require the integration of molecular and cellular techniques with data analysis packages to evaluate high throughput evidence from microscopy and flow cytometry. It is also necessary to develop new equipment or protocols for long‐term live cell tracking or high‐resolution microscopy beyond new molecular reporters, allowing the chronic study of live cells. Combining evidence from these diverse sources can transform the field, enhancing our comprehension of how SnCs acts on human health and extending beyond the advancement of more effective and specific senotherapies.

## AUTHOR CONTRIBUTIONS

T.C.B.: Writing and reviewing the manuscript; preparation of Tables S1 to S6; J.E.V.: Writing and reviewing the manuscript; preparation of Figure S1; A.O.S.: Writing and reviewing the manuscript; preparation of Table S7; L.R.F.: Writing and reviewing the manuscript; E.C.F.C.: Manuscript conception; writing and reviewing the manuscript; preparation of Figures 1, 2, 3 and Table 1.

## CONFLICT OF INTEREST STATEMENT

No conflict of interest.

## Supporting information


Figure S1

File S1

Table S1

Table S2

Table S3

Table S4

Table S5

Table S6

Table S7
