# Supplementary figures and images for: Subcellular structure, heterogeneity, and plasticity of senescent cells
Source: Aging Cell. 2024 Mar 30;23(4):e14154. doi: 10.1111/acel.14154 (PMC11019148; doi:10.1111/acel.14154)

Figure S1

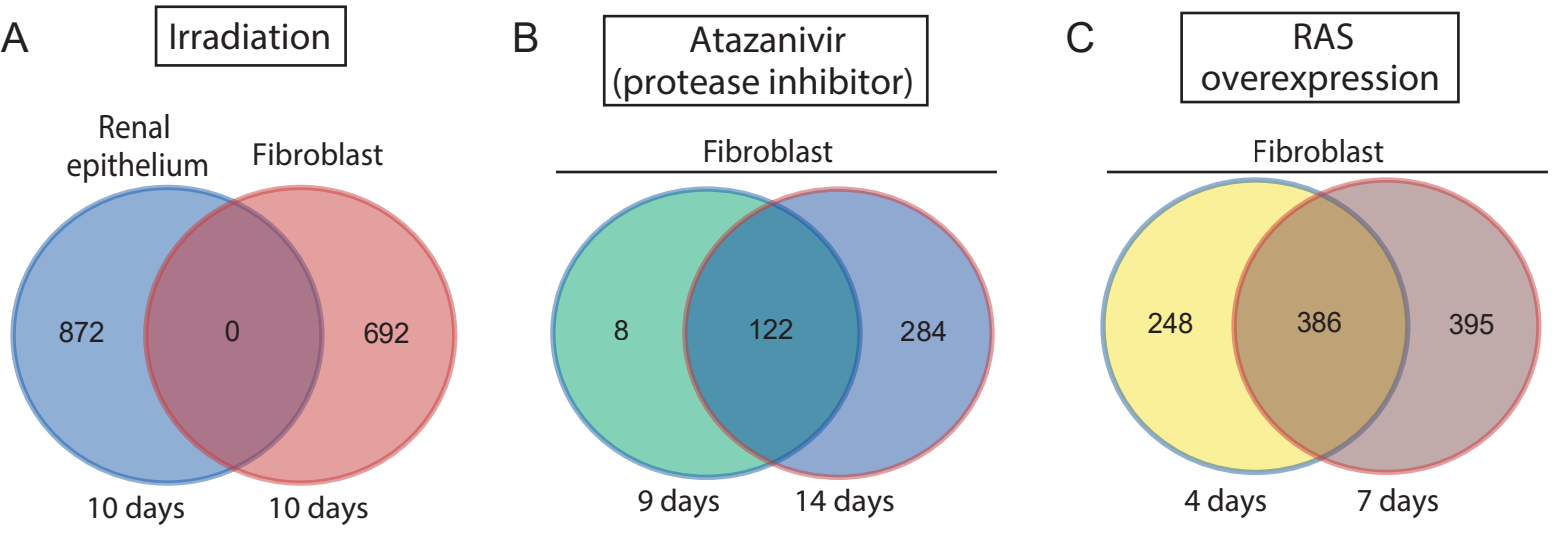

Supplement: Supplementary file 1 — Figure S1 [file ACEL-23-e14154-s003.pdf]
