# Supplementary material for: Subcellular structure, heterogeneity, and plasticity of senescent cells
Source: Aging Cell. 2024 Mar 30;23(4):e14154. doi: 10.1111/acel.14154 (PMC11019148; doi:10.1111/acel.14154)
Supplement: Supplementary file 2 — File S1 [file ACEL-23-e14154-s009.docx]

**Supplementary file 1 / Box 1 - Senescence markers**

Despite some specificities and the heterogeneity of SnCs, there are several cellular and molecular markers commonly used in the investigation of cellular senescence, including:

**Changes in cell morphology:** cells became flattened and enlarged (due to persistent mTOR activity), which seems to be involved in impaired cell proliferation, the establishment of the senescent state, and functional decline of SnCs. *In vitro*, cells may have protrusions, but this feature is difficult to see in vivo. Applicability: *in vitro;* there is evidence showing cellular enlargement *in vivo*.

**Changes in nuclear morphology and organization:** nuclear enlargement, reduction of Lamin-B1, formation of senescence-associated heterochromatin foci (SAHF). Formation of γ-H2A.X foci in replicative senescence and DNA damage-induced senescence. Telomere shortening in replicative senescence. Applicability: *in vitro* and *in vivo*.

**Lack of proliferative markers:** lack of Ki67 staining or BrdU/EdU incorporation. Applicability: *in vitro* and *in vivo*.

**Increased activity of the senescence-associated beta-galactosidase acid (SA-β-gal) enzyme:** senescence-associated beta-galactosidase (SA-β-gal) corresponds to the lysosomal beta-galactosidase, which is increased in SnCs. Applicability: *in vitro* and *in vivo*.

**Molecular markers:** in general, senescence is triggered due to the activation of DNA damage response (DDR) pathway, starting with the activation of γ-H2AX. This triggers the ATM/ATR-Chk1/2-TP53 pathway. Notwithstanding, other intracellular pathways can be activated (see Figure 1). The main molecular effectors of senescence are p21^CIP1/WAF1^ (*CDKN1A*) and p16^INK4a/Arf^ (*CDKN2A*). p16^INK4a/Arf^ is the predominant senescence marker also for *in vivo* studies, while p21 is important for senescence induction but not for its maintenance). In specific conditions, other CDKi can execute cellular senescence, including p27, p15INK^4b^ or p14^Arf^ (p19^Arf^ in mice). Applicability: *in vitro* and *in vivo*.

**Senescence-associated secretory phenotype (SASP):** SnCs secrete a range of factors, including cytokines, chemokines, growth factors, and matrix metalloproteinases which collectively make up the SASP. It also includes extracellular vesicles. Applicability: *in vitro* and *in vivo (unspecific)*.

The choice of markers to assess cellular senescence depends on the specific experimental setup and the cell types being studied, since these markers might not be present in all SnCs. It is consensus that a single marker is not enough to characterize senescence. Currently, The International Cell Senescence Association (ICSA) suggests a three-step strategy that involves the analysis of: (1) a lysosome marker (e.g. the activity of senescence-associated acidic beta galactosidase - SA-β-Gal, or lypofucsin), (2) molecular markers (e.g. p16, p21, Lamin-B1, or proliferation marker), and (3) marker of specific types of senescence (e.g. SASP, DNA damage, or intracellular pathways) (Gorgoulis et al, 2019). Finally, as discussed along the review, several differentially expressed genes have been described in SnCs, mainly cell surface proteins, many of which can be assessed. Likewise, increased levels of pro-survival or anti-apoptotic proteins can also contribute on the investigation of cellular senescence.
