# Supplementary material for: Subcellular structure, heterogeneity, and plasticity of senescent cells
Source: Aging Cell. 2024 Mar 30;23(4):e14154. doi: 10.1111/acel.14154 (PMC11019148; doi:10.1111/acel.14154)
Supplement: Supplementary file 3 — Table S1 [file ACEL-23-e14154-s004.docx]

**Supplementary Table 1 - Plasma membrane changes in SnCs.**

| **Senescence inducer** | **Cell Model** | **Senescence markers** | **Findings of SnCs plasma membrane** | **Type of data** | **Ref** |
| --- | --- | --- | --- | --- | --- |
| RS | HS68 cell line (fibroblasts) | SA β-Gal (microscopy) | ↓ membrane fluidity, ↓ cholesterol and ceramide-rich lipid rafts; ↑ hydrophobicity and GM1 ganglioside levels | SEP and SSC | (Wi et al., 2021) |
| RS | TIG-1 cell line (fibroblasts) | SA β-Gal (microscopy) | ↓ cholesterol | SEP and SSC | (Nakamura et al., 2003) |
| DDIS | SK-MEL-103 (melanoma) | SA β-Gal (microscopy) | ↑ LAMP1 and LAMP2 | SEP and SSC | (Rovira et al., 2022) |
| DDIS, OIS, RS | Primary fibroblasts | p16, Ki67 (flow cytometry), SASP | ↑ HLA-E | SEP and SSC | (Pereira et al., 2019) |
| RS | Primary fibroblasts | SA β-Gal (microscopy), cell morphology, BrdU, SASP, p16 | ↑ ACKR3 (CXCL12 receptor) | SEP and SSC | (Takaya et al., 2022) |
| RS | HS68 cells | Cell morphology, SA β-Gal (not showed) | More diffuse pattern of caveolin in SnCs | SEP and SSC | (Wheaton et al., 2001) |

DDIS, DNA damage-induced senescence; ICC, immunocytochemistry; OIS, oncogene-induced senescence; RS, replicative senescence; SASP, senescence-associated secretory phenotype; SEP, senescence-enriched population; SSC, single senescent cells; ↑, increased; ↓, decreased.

Supporting References

Nakamura, M., Kondo, H., Shimada, Y., Waheed, A. A., & Ohno‐Iwashita, Y. (2003). Cellular aging‐dependent decrease in cholesterol in membrane microdomains of human diploid fibroblasts. Experimental Cell Research, 290(2), 381–390. https://doi.org/10.1016/s0014‐4827(03)00343‐4
