# Supplementary material for: Subcellular structure, heterogeneity, and plasticity of senescent cells
Source: Aging Cell. 2024 Mar 30;23(4):e14154. doi: 10.1111/acel.14154 (PMC11019148; doi:10.1111/acel.14154)
Supplement: Supplementary file 4 — Table S2 [file ACEL-23-e14154-s002.docx]

**Supplementary Table 2 - Cytoskeleton changes in SnCs.**

| **Senescence inducer** | **Cell Model** | **Senescence markers** | **Findings of SnCs cytoskeleton** | **Type of data** | **Ref** |
| --- | --- | --- | --- | --- | --- |
| RS | TIG101 cells (fibroblasts cell line) | Microscopy (cell morphology), PCNA (telomeres length) | ↑ vimentin | SEP and SSC | (Nishio et al., 2001) |
| RS | TIG101 cells | Microscopy (cell morphology), PCNA (telomeres length) | ↑ vimentin | SEP and SSC | (Nishio & Inoue, 2005) |
| Sodium butyrate or  lopinavir | LLC-PK1 cell line (epithelial cells) | Microscopy (cell morphology) | ↑ microtubule stability and α-tubulin acetylation (lys40);  ↓ HDAC6 and Rock1; reorganization of actin filaments, mainly in cell cortex | SEP and SSC | (Moujaber et al., 2019) |
| DDIS | Cancer cell lines: MCF7 (breast), U2OS (osteosarcoma), U87 (glioblastoma), HT29 (colorectal), A549 (lung) | SA β-Gal | Altered localization of nucleoskeleton-cytoskeleton ligand complex (LINC); altered pattern of nuclear emerin and LaminA/C | SEP | (Svobodová et al., 2020) |
| DDIS | NCI-H460 (lung cancer), HCT116 (colorectal cancer) | Microscopy (cell morphology), SA β-Gal, p21 | ↑ vimentin degradation | SEP and SSC | (Hammer et al., 2022) |
| RS | HUVEC cell line (epithelial cells) | Microscopy (cell morphology), SA β-Gal | ↑ Myosin 1b (Myo1b) | SEP and SSC | (Yu et al., 2023) |

DDIS, DNA damage-induced senescence; ICC, immunocytochemistry; OIS, oncogene-induced senescence; RS, replicative senescence; SASP, senescence-associated secretory phenotype; SEP, senescence-enriched population; SSC, single senescent cells; ↑, increased; ↓, decreased.

Supporting references:

Svobodová Kovaříková, A., Bártová, E., Kovařík, A., & Lukášová, E. (2020). Spatiotemporal Mislocalization of nuclear membrane‐associated proteins in γ‐irradiation‐induced senescent cells. Cells, 9(4), 999. https://doi.org/10.3390/cells9040999
