# Supplementary material for: Subcellular structure, heterogeneity, and plasticity of senescent cells
Source: Aging Cell. 2024 Mar 30;23(4):e14154. doi: 10.1111/acel.14154 (PMC11019148; doi:10.1111/acel.14154)
Supplement: Supplementary file 5 — Table S3 [file ACEL-23-e14154-s008.docx]

**Supplementary Table 3 - Mitochondrial changes in SnCs.**

| **Senescence inducer** | **Cell Model** | **Senescence markers** | **Findings of SnCs mitochondrial network** | **Type of data** | **Ref** |
| --- | --- | --- | --- | --- | --- |
| DDIS, OIS | Primary fibroblasts, primary CAFs, MRC-5 cell line (fibroblasts) | SA β-Gal, microscopy, p16 and TP53 | Mitochondrial depolarization by ↓ Ca^2+^ transfer to mitochondria and ↑ ROS production | SEP and SSC | (Farfariello et al., 2022) |
| RS, DDIS, OIS | HF043 (primary fibroblast), IMR90 cell line (fibroblasts) | SA β-Gal, Microscopy (cell morphology), IL6 secretion and p21 (protein) levels | Mitochondria transfer between SnCs or between SnCs and non-SnCs cells via membrane nanotubes | SEP and SSC | (Walters & Cox, 2021) |
| DDIS | B16-F1 cell line (epithelial cells) | SA β-Gal, Microscopy (cell morphology), TP53 and p21 (protein) | ↑ mitochondrial respiration and mass; ↑ mitochondrial fusion events with elongated mitochondria; ↑ PGC-1α, TFAM and MFN1 | SEP and SSC | (Martínez et al., 2019) |
| RS | PANC-1 cell line (pancreatic cancer) | SA β-Gal, Microscopy (cell morphology) - no available | ↓ carnitine palmitoyltransferase 1C activity (CPT1) | SEP and SSC | (Wang et al., 2018) |
| DDIS | Cancer cell lines NCI-H460 (lung), HCT116 (colorectal) | Microscopy (cell morphology), SA β-Gal, p21 | ↑ cytoplasmic mitochondrial serine protease HTRA2 | SEP and SSC | (Hammer et al., 2022) |

DDIS, DNA damage-induced senescence; ICC, immunocytochemistry; OIS, oncogene-induced senescence; RS, replicative senescence; SASP, senescence-associated secretory phenotype; SEP, senescence-enriched population; SSC, single senescent cells; CAFs, cancer-associated fibroblasts; ↑, increased; ↓, decreased.
