# Supplementary material for: Subcellular structure, heterogeneity, and plasticity of senescent cells
Source: Aging Cell. 2024 Mar 30;23(4):e14154. doi: 10.1111/acel.14154 (PMC11019148; doi:10.1111/acel.14154)
Supplement: Supplementary file 6 — Table S4 [file ACEL-23-e14154-s001.docx]

**Supplementary Table 4 – Alterations in lysosomes and autophagy in SnCs.**

| **Senescence inducer** | **Cell Model** | **Senescence markers** | **Findings of SnCs lysosomes and autophagy** | **Type of data** | **Ref** |
| --- | --- | --- | --- | --- | --- |
| TIS, OIS, RS | HEK293T cell line (epithelial), MRC5 and  IMR90 | Microscopy (cell morphology), p16, SASP (IL-6) | ↑ lysosomal mass; ↑ permeability in lysosomal membrane, ↑ pH and ↓ proteolytic activity | SEP and SSC | (Curnock et al., 2023) |
| RS | IMR90, Detroit 551 and BJ-hTERT cell lines (fibroblasts); HEK293T and AD293 (epithelial cell lines) | SA β-Gal | ↑ lysosomal content. | SEP and SSC | (Liao et al., 2022) |
| DDIS | MCF7 and 4226 cell lines (breast cancer) | Microscopy (cell morphology), SA β-Gal; SASP | Lysosomal breakdown of engulfed cells | SEP and SSC | (Tonnessen-Murray et al., 2019) |
| Microtubule inhibition (Vincristine) | Cancer cell lines: HeLa (cervical), MCF7 (breast) | Microscopy (cell morphology) | ↑ size and number of lysosomes, lysosomal membrane permeabilization | SEP and SSC | (Groth-Pedersen et al., 2007) |
| RS | MRC5 cell line (fibroblast) | SA β-Gal, microscopy (cell morphology) | ↑ number and area of autophagic vacuole | SEP and SSC | (Gerland et al., 2003) |
| RS and OIS | IMR90 cell line (fibroblast), primary melanocytes | SA β-Gal, cell morphology; p16 and cyclin A (protein) | ↑ autophagy and nucleophagy by nuclear ‘bubbling’ | SEP and SSC | (Ivanov et al., 2013) |
| RS and OIS | Primary fibroblasts | SA β-Gal, microscopy (cell morphology), p16, ki67 | ↑ ferritin H, suggesting slower protein turnover; ↓ formation of LC3-II | SEP and SSC | (Ott et al., 2016) |
| RS | HUVECs cell line (epithelial) | Microscopy (cell morphology), SA β-Gal | ↑ intracellular Ca^2+^, compromising autophagosome-lysosome assembly (i.e. impaired autophagic flux) | SEP and SSC | (Yu et al., 2023) |
| DDIS, OIS | Cell lines: NIH 3T3 and IMR90 (fibroblasts), SK-MEL-103 (melanoma), Huh7 (liver cancer), U2OS and SAOS-2 (osteosarcoma) | SA β-Gal, Microscopy (cell morphology) | ↑ TFEB mRNA, ↑ macroautophagy and chaperone-mediated autophagy | SEP and SSC | (Rovira et al., 2022) |
| OIS, OIS, DDIS | IMR90 cell line (fibroblasts), HEK293T cell line (epithelial) and primary fibroblasts | Microscopy (cell morphology), p16 | Nuclear LC3 interaction with Lamin B1. Nuclear bubbling and formation of cytoplasmic chromatin fragments (CCFs) | SEP and SSC | (Dou et al., 2015) |
| OIS | NIH3T3 and MRC-5 cell lines (fibroblasts) | SA β-Gal | Defective DNA degradation by autolysosome and CCFs accumulation | SEP and SSC | (Han et al., 2020) |

DDIS, DNA damage-induced senescence; ICC, immunocytochemistry; OIS, oncogene-induced senescence; RS, replicative senescence; SASP, senescence-associated secretory phenotype; SEP, senescence-enriched population; SSC, single senescent cells; ↑, increased; ↓, decreased.

Supporting references:

Yu, Y., Ren, Y., Li, Z., Li, Y., Zhang, Y., Gui, R., Gui, R., Cui, Y., Qian, L., & Xiong, Y. (2023). Myo1b promotes premature endothelial senescence and dysfunction via suppressing autophagy: Implications for vascular aging. Oxidative Medicine and Cellular Longevity, 2023, 4654083. https://doi.org/10.1155/2023/4654083
