# Supplementary material for: Subcellular structure, heterogeneity, and plasticity of senescent cells
Source: Aging Cell. 2024 Mar 30;23(4):e14154. doi: 10.1111/acel.14154 (PMC11019148; doi:10.1111/acel.14154)
Supplement: Supplementary file 7 — Table S5 [file ACEL-23-e14154-s007.docx]

**Supplementary Table 5 - Nuclear changes in SnCs.**

| **Senescence inducer** | **Cell Model** | **Senescence markers** | **Findings of SnCs nuclei** | **Type of data** | **Ref** |
| --- | --- | --- | --- | --- | --- |
| RS | Primary fibroblasts; primary keratinocytes; | Microscopy (cell morphology), SA β-Gal, ki67, p53 | ↓ Lamin B1 | SSC and SEP | (Dreesen et al., 2013) |
| DDIS | Primary keratinocytes | Microscopy (cell morphology), SA β-Gal, p16, p21, p19 | ↓ Lamin B1 | SEP and SSC | (Wang et al., 2017) |
| RS, OIS, DDIS | IMR90 (fibroblasts), HEK293T and primary BJ fibroblasts | Microscopy (cell morphology), p16 | Lamin B1 interacts with nuclear LC3 and CCFs that co-localize with autophagosomes | SEP and SSC | (Dou et al., 2015) |
| RS and OIS | IMR90 cell line (fibroblasts), human epidermal melanocytes | SA β-Gal, cell morphology, p16 and cyclin A | ↓ histone by nuclei ‘bubbling’ and nucleophagy | SEP and SSC | (Ivanov et al., 2013) |
| OIS | NIH3T3 cell line (fibroblasts), MRC-5 cell line (fibroblasts) | SA β-Gal | Compromised nuclear envelope; nuclear blebbing and CCFs accumulation; ↓ total histone content | SEP and SSC | (Han et al., 2020) |
| DDIS | Cancer cell lines: U87 (glioblastoma), C6 (astrocytoma), HeLa (cervical) and HCT116 (colorectal) | SA-β-gal, nuclear morphometry | Nuclear enlargement in SA-β-gal positive cells | SEP and SSC | (Filippi-Chiela et al., 2012) |
| RS | Hutchinson-Gilford fibroblasts | SA-β-gal, nuclear morphometry | Clustering of Nuclear Pore Complex (NPC) in dysmorphic nuclei | SEP and SSC | (Röhrl et al., 2021) |

DDIS,DNA Damage-induced senescence; ICC, immunocytochemistry; OIS, oncogene-induced senescence; RS, replicative senescence; SASP, senescence-associated secretory phenotype; SEP, senescence-enriched population; SSC, single senescent cells; ↑, increased; ↓, decreased.

Supporting reference:

Dreesen, O., Chojnowski, A., Ong, P. F., Zhao, T. Y., Common, J. E., Lunny, D., Lane, E. B., Lee, S. J., Vardy, L. A., Stewart, C. L., & Colman, A. (2013). Lamin B1 fluctuations have differential effects on cellular proliferation and senescence. The Journal of Cell Biology, 200(5), 605–617. https://doi.org/10.1083/jcb.201206121

Ivanov, A., Pawlikowski, J., Manoharan, I., van Tuyn, J., Nelson, D. M., Rai, T. S., . . . Adams, P. D. (2013). Lysosome-mediated processing of chromatin in senescence. J Cell Biol, 202(1), 129-143. doi:10.1083/jcb.201212110
