# Supplementary material for: Subcellular structure, heterogeneity, and plasticity of senescent cells
Source: Aging Cell. 2024 Mar 30;23(4):e14154. doi: 10.1111/acel.14154 (PMC11019148; doi:10.1111/acel.14154)
Supplement: Supplementary file 8 — Table S6 [file ACEL-23-e14154-s006.docx]

**Supplementary Table 6 –** **Changes in Golgi apparatus and endoplasmic reticulum (ER) in SnCs.**

| **Senescence inducer** | **Cell Model** | **Senescence markers** | **Findings of SnCs Golgi apparatus and ER** | **Type of data** | **Ref** |
| --- | --- | --- | --- | --- | --- |
| RS | WI38 and IMR90 cell lines (fibroblasts), HeLa (cervical cancer) | Cell morphology, p21 | ↑ γ11 subunit of the G protein leading to the dispersion of the Golgi apparatus | SEP | (Cho et al., 2011) |
| RS | TIG-1 cell line (fibroblasts) and HEK293 (epithelial) | SA-β-Gal | ↓ ATP6V0A2 associated with glycosylation changes and dispersion of the Golgi apparatus | SEP and SSC | (Udono et al., 2015) |
| DDIS, OIS, OIS. | Primary fibroblasts, MRC-5 cell line (fibroblasts), CAFs | SA β-Gal, cell morphology, p16 and p53 | ↓ Ca^2+^ transfer between endoplasmic reticulum and mitochondria | SEP and SSC | (Farfariello et al., 2022) |

DDIS, DNA damage-induced senescence; ICC, immunocytochemistry; OIS, oncogene-induced senescence; RS, replicative senescence; SASP, senescence-associated secretory phenotype; SEP, senescence-enriched population; SSC, single senescent cells; CAFs, cancer-associated fibroblasts; ↑, increased; ↓, decreased.
