# Supplementary material for: Subcellular structure, heterogeneity, and plasticity of senescent cells
Source: Aging Cell. 2024 Mar 30;23(4):e14154. doi: 10.1111/acel.14154 (PMC11019148; doi:10.1111/acel.14154)
Supplement: Supplementary file 9 — Table S7 [file ACEL-23-e14154-s005.docx]

**Supplementary Table 7 – Pro-survival pathways in SnCs**.

| **Pro-survival player** | **Senotherapy** | **Main Outcome** | **Other relevant molecular changes** | **Model** | **Ref** |
| --- | --- | --- | --- | --- | --- |
| **TP53/p21 pathway** | |  |  |  |  |
| TP53 | Inactivation by small t-antigen | TP53 inactivation reverted RS in a p16-dependent manner. in cells with low p16. | Decrease in p16 | Human fibroblast | (Beauséjour et al., 2003) |
| TP53 | No | CDK2AP1 KD-induced senescence increases DNA damage, upregulating TP53 and its downstream targets p21, BAX, and PUMA. | Increase in p21, BAX and PUMA | Human fibroblast | (Alsayegh, et al., 2015) |
| p21 | KD (iRNA) | KD of p21 in DDIS induce cell death through ATM and NF-kb activation, in a caspase- and JNK-dependent manner. | Increase in CDK-1, Cyclin A2, Smad-7, and TNF-a | Human and mouse fibroblast, and NSCL carcinoma | (Yosef et al., 2017) |
| TP53/p21 | No | IR-induced senescence can prime fibroblast cells to trigger apoptosis. However, cell death was prevented by FOXO4, which modulates p21 through TP53 to induce senescence. | Increase in PUMA, BIM, and FOXO4. Decrease in BCL-2 | Human fibroblast | (Baar et al., 2017) |
| **Bcl-2 pathway** | |  |  |  |  |
| Bcl-2 family | No | Temperature-activated senescence induced BCL-2 and BAX increasing. | Increase of p21 and hypophospho-Rb | Rat Embryo fibroblast | (Baar et al., 2017) |
| Bcl-2 | Pharmacological | SnCs presented upregulation of Bcl-2, BID, and BAX, and downregulation of Mcl-1, Bad, Bak, and Bim. The navitoclax sensitized SnCs to die. |  | Human kidney epithelial cell | (Yang et al., 2022) |
| Bcl-2 | Overexpression | Blocking apoptosis by upregulation of Bcl-2 increased levels of senescence. |  | Human leukemia cell | (Drullion et al., 2012) |
| Bcl-2, BAX | no | RS showed both constitutive and inducible increases in Bcl-2 expression and a BAX decrease, supporting an apoptosis-resistant phenotype. | Increase in p21 and p16 | Human Fibroblast | (Sanders et al., 2013) |
| Bcl-2, Bcl-xL | Pharmacological | Treatment with Navitoclax sensitized SnCs to die. |  | Alveolar Epithelial Cell | (Pan et al., 2017) |
| Bcl-2, Bcl-xL | Pharmacological | Treatment with Navitoclax, sensitized SnCs to die in vivo and in vitro. | Decrease in p16 and p21 | 8-week-old C57bL/6j mice | (Su et al., 2023) |
| Bcl-2, Bcl-xL, Bcl-w | No | Three different ways to induce senescence in fibroblasts result in the upregulation of BCL-W, BCL-xL, and BCL-2. | Increase in p16 and p21 | Human and mouse fibroblast | (Yosef et al., 2016) |
| Bcl-2, Bcl-xL, Bcl-w | Pharmacological | Senescent fibroblasts were sensitized to die with ABT-737, a potent inhibitor of BCL-2, BCL-xL, and BCL-W. |  | Human and mouse fibroblast | (Yosef et al., 2016) |
| Bcl-xL, Bcl-w | KD (iRNA) | Combining the inhibition of BCL-xL and BCL-w, the DNA damage-induced SnCs were sensitized to die. |  | Human and mouse fibroblast | (Storer et al., 2013) |
| Bcl-xL | Pharmacological inhibition | Two relatively specific BCL-xL inhibitors, A1331852 and A1155463, can eliminate senescent HUVEC and IMR90 cells but not senescent preadipocytes. |  | Human  endothelial cells and fibroblast | (Zhu et al., 2017) |
| Bcl-xL | Dowregulation | BCL-xL inhibition induced apoptosis in response to BETi even after BETi-induced senescence had already occurred. |  | Human Breast Cancer cell | (Gayle et al., 2019) |
| Bcl-xL | Pharmacological or genetic downregulation | Downregulation of Bcl-xL (Navitoclax or A-1331852) reduced the cell viability through apoptosis. On the other hand, the Bcl-2 inhibitor (Venetoclax) and Mcl-1 inhibitor (S63845) did not impact the viability of SnCs. |  | Pilocytic Astrocitoma cell | (Selt et al., 2023) |
| Bid, BAX | Pharmacological inhibition | The BH3 mimetic A-1331852 induced caspase-dependent SnC death, releasing the binding between BCL-xL and tBID and dissociating the binding between BCL-xL and BAX. |  | Human lung carcinoma | (Wu et al., 2022) |
| BAX | No | Senescence induced by irradiation plus MDM-2 inhibitor induced TP53 accumulation, followed by an increase in p21 and BAX. | Accumulation of TP53 and increase in p21. | Melanoma and sarcoma cell | (Werner et al., 2015) |
| BIM | Downregulation | Block apoptosis by decreasing BIM increased levels of senescence. |  | Human leukemia | (Drullion et al., 2012) |

Supporting references:

Beauséjour, C. M., Krtolica, A., Galimi, F., Narita, M., Lowe, S. W., Yaswen, P., & Campisi, J. (2003). Reversal of human cellular senescence: Roles of the p53 and p16 pathways. The EMBO Journal, 22(16), 4212–4222. https://doi.org/10.1093/emboj/cdg417

Dreesen, O., Chojnowski, A., Ong, P. F., Zhao, T. Y., Common, J. E., Lunny, D., Lane, E. B., Lee, S. J., Vardy, L. A., Stewart, C. L., & Colman, A. (2013). Lamin B1 fluctuations have differential effects on cellular proliferation and senescence. The Journal of Cell Biology, 200(5), 605–617. https://doi.org/10.1083/jcb.201206121

Drullion, C., Tregoat, C., Lagarde, V., Tan, S., Gioia, R., Priault, M., Djavaheri‐Mergny, M., Brisson, A., Auberger, P., Mahon, F. X., & Pasquet, J. M. (2012). Apoptosis and autophagy have opposite roles on imatinib‐induced K562 leukemia cell senescence. Cell Death & Disease, 3(8), e373. https://doi.org/10.1038/cddis.2012.111

Gayle, S. S., Sahni, J. M., Webb, B. M., Weber‐Bonk, K. L., Shively, M. S., Spina, R., Bar, E. E., Summers, M. K., & Keri, R. A. (2019). Targeting BCL‐xL improves the efficacy of bromodomain and extra‐terminal protein inhibitors in triple‐negative breast cancer by eliciting the death of senescent cells. The Journal of Biological Chemistry, 294(3), 875–886. https://doi.org/10.1074/jbc.RA118.004712

Idda, M. L., McClusky, W. G., Lodde, V., Munk, R., Abdelmohsen, K., Rossi, M., & Gorospe, M. (2020). Survey of senescent cell markers with age in human tissues. Aging, 12(5), 4052–4066. https://doi.org/10.18632/aging.102903

Pan, J., Li, D., Xu, Y., Zhang, J., Wang, Y., Chen, M., Lin, S., Huang, L., Chung, E. J., Citrin, D. E., Wang, Y., Hauer‐Jensen, M., Zhou, D., & Meng, A. (2017). Inhibition of Bcl‐2/xl with ABT‐263 selectively kills senescent type II pneumocytes and reverses persistent pulmonary fibrosis induced by ionizing radiation in mice. International Journal of Radiation Oncology, Biology, Physics, 99(2), 353–361. https://doi.org/10.1016/j.ijrobp.2017.02.216

Sanders, Y. Y., Liu, H., Zhang, X., Hecker, L., Bernard, K., Desai, L., Liu, G., & Thannickal, V. J. (2013). Histone modifications in senescence‐associated resistance to apoptosis by oxidative stress. Redox Biology, 1(1), 8–16. https://doi.org/10.1016/j.redox.2012.11.004

Su, W., Hu, Y., Fan, X., & Xie, J. (2023). Clearance of senescent cells by navitoclax (ABT263) rejuvenates UHMWPE‐induced osteolysis. International Immunopharmacology, 115, 109694. https://doi.org/10.1016/j.intimp.2023.109694

Werner, L. R., Huang, S., Francis, D. M., Armstrong, E. A., Ma, F., Li, C., Iyer, G., Canon, J., & Harari, P. M. (2015). Small molecule inhibition of MDM2‐p53 interaction augments radiation response in human tumors. Molecular Cancer Therapeutics, 14(9), 1994–2003. https://doi.org/10.1158/1535‐7163.MCT‐14‐1056‐T

Wu, G., Li, X., Zhan, Y., Fan, X., Xu, L., Chen, T., & Wang, X. (2022). BID‐ and BAX‐mediated mitochondrial pathway dominates A‐1331852‐induced apoptosis in senescent A549 cells. Biochemical and Biophysical Research Communications, 627, 160–167. https://doi.org/10.1016/j.bbrc.2022.08.023

Yang, Y., Mihajlovic, M., Valentijn, F., Nguyen, T. Q., Goldschmeding, R., & Masereeuw, R. (2022). A human conditionally immortalized proximal tubule epithelial cell Line as a novel model for studying senescence and response to Senolytics. Frontiers in Pharmacology, 13, 791612. https://doi.org/10.3389/fphar.2022.791612
